# Aphasia severity mediates the relationship between attention and sentence comprehension

**DOI:** 10.1080/02687038.2026.2671291

**Published:** 2026-05-18

**Authors:** Emily J. Lenz, Arianna N. LaCroix

**Affiliations:** Department of Speech, Language, and Hearing Sciences, Purdue University, West Lafayette, IN, USA

**Keywords:** Aphasia, attention, orienting, executive control, sentence comprehension

## Abstract

**Background:**

Prior studies report mixed associations between attention and language in people with aphasia (PWA), raising the possibility that their relationship may be influenced by another factor, such as aphasia severity. We examined this possibility by testing whether alerting, orienting, and executive control attention vary as a function of aphasia severity and whether severity statistically mediates their association with sentence comprehension.

**Methods:**

In a retrospective analysis combining data from two prior studies, 58 individuals with aphasia, ranging from latent to severe, and 21 neurotypical controls completed the Attention Network Test and a sentence-picture matching task. Group differences in attention were assessed using ANOVAs, and linear mixed-effects models evaluated the presence of each attentional effect within groups. Correlations and structural equation modeling examined associations among attention, aphasia severity, and sentence comprehension.

**Results:**

All three attention types were present in the control and mild aphasia groups; only orienting and executive control were observed in the latent group, and only executive control in the moderate and severe groups. Orienting and executive control performance declined with increasing aphasia severity, whereas alerting did not differ by severity. Stronger orienting and executive control were associated with more efficient sentence comprehension, but this relationship was statistically mediated by aphasia severity.

**Conclusions:**

These findings suggest that associations between domain-general attention and sentence comprehension are best understood within the broader context of aphasia severity and highlight the need for future research clarifying the directionality of this relationship.

## Introduction

Aphasia affects approximately one-third of stroke survivors and creates major barriers to communication, independence, and quality of life. Beyond language impairments, up to 80% of people with aphasia (PWA) experience additional cognitive deficits in the first-year post-stroke (El Hachioui et al., 2014), among which domain-general attention difficulties are particularly consequential, as they contribute to poorer language performance (LaCroix, Blumenstein, et al., 2020; Murray, 2012; Murray et al., 1997; Varkanitsa et al., 2023; Villard & Kiran, 2017).

The attentional subsystems model defines attention as the coordinated function of three interacting networks: alerting (achieving and maintaining a state of readiness), orienting (shifting attention toward relevant stimuli), and executive control (resolving conflict among competing stimuli or responses while completing goal-directed behavior) (Petersen & Posner, 2012; Posner & Petersen, 1990). Although well-established in the neurotypical literature (de Souza Almeida et al., 2021), this framework has only recently been applied to the study of attention in aphasia (Dash et al., 2020; Huang et al., 2022; LaCroix, Tully, et al., 2020; Mohapatra & Dash, 2023).

The attentional subsystems model offers two potential advantages for studying attention in aphasia. First, the Attention Network Test assesses alerting, orienting, and executive control efficiently using a single cued-flanker task (Fan et al., 2002). Because the task is largely nonverbal, it minimizes linguistic demands that often confound cognitive assessment in aphasia, thereby increasing the interpretability of attentional performance. Second, the model is grounded in well-characterized neuroanatomical systems. Alerting has been linked to a right-lateralized frontoparietal system and noradrenergic input from the locus coeruleus; orienting is supported by a bilateral dorsal frontoparietal network (including the intraparietal sulcus and frontal eye fields) and a ventral reorienting system; and executive control relies on cingulo-opercular and dorsolateral prefrontal systems (Corbetta & Shulman, 2002; Markett et al., 2021; Petersen & Posner, 2012; Rinne et al., 2013; Xiao et al., 2016). Contemporary network-level accounts of aphasia further emphasize that behavioral impairments arise from disruption to large-scale functional networks rather than damage to isolated cortical regions (Kiran et al., 2019; Wilmskoetter et al., 2022). From this perspective, the efficiency of alerting, orienting, and executive control likely reflects the integrity and connectivity of these attention hubs and their interaction with the language network. Consequently, different lesion patterns may produce distinct attentional deficits not simply due to focal injury, but because disruption to critical network hubs alters communication within and between these networks.

Studies applying the subsystems model to aphasia have reported reduced efficiency in the alerting and orienting networks compared with controls (Dash et al., 2020; Huang et al., 2022; LaCroix, Tully, et al., 2020; Mohapatra & Dash, 2023). These findings align with earlier evidence that PWA have difficulty sustaining attention (Korda & Douglas, 1997; Laures, 2005) and orienting to salient stimuli (Petry et al., 1994; Robin & Rizzo, 1989; Villard & Kiran, 2015). Results for executive control are more mixed: some studies find no differences between PWA and controls (Dash et al., 2020; LaCroix, Tully, et al., 2020; Mohapatra & Dash, 2023), while others document impaired performance in PWA (Huang et al., 2022). The latter finding is consistent with the broader selective attention literature, which also reports executive control difficulties following a stroke (Erickson et al., 1996; Heuer & Hallowell, 2015; Murray, 2012; Pompon et al., 2015; Schumacher et al., 2022; Simic et al., 2020; Villard & Kiran, 2017).

Findings across studies suggest that the three attentional subsystems are differentially vulnerable in aphasia, yet the functional consequences of these impairments for language are not well understood. Nevertheless, accumulating evidence indicates that alerting, orienting, and executive control may contribute to language processing in distinct ways. For instance, research in neurotypical older adults shows that reduced alertness compromises sentence processing (LaCroix, Blumenstein, et al., 2020), possibly because individuals are less prepared to process incoming acoustic information. Such decreased readiness may also contribute to phonological, lexical, semantic, and repetition deficits in aphasia (Huang et al., 2022; Pérez Naranjo et al., 2018). Difficulty orienting to critical information, such as prosodic cues marking clause structure, has been shown to disrupt sentence comprehension (LaCroix, Blumenstein, et al., 2020; Oakley, 2009; Schafer, 1997), with orienting deficits also linked to poorer repetition skills (Huang et al., 2022). Executive control and selective attention are also important, as difficulties suppressing distracting information result in poorer sentence comprehension, particularly in noisy environments (Fitzhugh et al., 2021; Huang et al., 2022; Kalbe et al., 2005; Villard & Kidd, 2019). Executive control deficits have been further linked to conversational challenges (Frankel et al., 2007) and difficulties retelling stories in quiet (Dutta et al., 2024) and noise (Harmon et al., 2024; Nelson et al., 2023).

While prior work suggests that variability in attentional processes may contribute to differences in language impairment across PWA, aphasia severity remains a central factor, as it reflects the overall magnitude of language disruption and is often the strongest predictor of post-stroke language outcomes, with greater severity associated with more extensive deficits and poorer treatment response (Fridriksson & Hillis, 2021; Kristinsson et al., 2023; Nakagawa et al., 2019; Plowman et al., 2012). Despite this, relatively few studies have directly examined how attention relates to aphasia severity, and the results from existing studies are mixed: some report that poorer attention is associated with more severe aphasia and greater language impairment (Fonseca et al., 2019; Lee et al., 2020; Meier et al., 2022; Murray, 2012; Schumacher et al., 2022), whereas others find no relationship (Gordon-Pershey & Wadams, 2017; Murray et al., 1997; Yao et al., 2020).

In this retrospective analysis combining data from two prior studies, we examined whether the presence and magnitude of attentional deficits vary as a function of aphasia severity and whether severity statistically mediates the relationship between attention and sentence comprehension performance. Aphasia severity was indexed using the Western Aphasia Battery-Revised (WAB-R; Kertesz, 2007). Sentence comprehension was assessed using a sentence – picture matching task, which was the only language measure, apart from the WAB-R, administered consistently across both datasets. WAB-R subscores were not used as language outcomes to avoid construct overlap and potential circularity, as aphasia severity was derived from the same instrument. We hypothesized that individuals with more severe aphasia would exhibit greater attention deficits than those with milder aphasia, both within- and between groups. Further, we predicted that aphasia severity would statistically mediate the relationship between attention and sentence comprehension, such that weaker attention would be associated with poorer sentence comprehension as a consequence of greater aphasia severity.

## Method

### Participants

Fifty-eight left hemisphere stroke survivors participated in this study (24 female). Participants ranged in age from 31 to 82 years (*M* = 59.47, s*d* = 10.02), were pre-morbidly right-handed, >6 months post-stroke, had normal or corrected to normal vision and hearing, and were native speakers of American English who denied premorbid diagnosis of neurological disease, head trauma, or psychiatric diagnosis. Aphasia type and severity were determined using the WAB-R. Overall, the sample included eight participants with latent aphasia (WAB-AQ > 93.8), 23 with mild aphasia, 21 with moderate aphasia, 3 with severe aphasia, and 3 with very severe aphasia. We combined the severe and very severe aphasia participants into a single group due to their small sample sizes; we refer to this group as the “severe” aphasia group hereafter. Table 1 provides a detailed description of the stroke participants, including demographic and study-specific information.

Twenty-one right-handed, American English speakers served as the control group (10 female). Control participants ranged in age from 24 to 77 years (*M* = 57.14, s*d* = 13.04) and were matched to the stroke group on age and education (*p* > .05). All participants provided written informed consent prior to participating. Study procedures were approved by the Midwestern University (IRB AZ1464) and Purdue University (IRB 2023-1067) Institutional Review Boards.

### Procedure

This study is a retrospective analysis combining data from two previous experiments involving left hemisphere stroke participants who all completed the WAB-R, Attention Network Test (ANT), and a sentence-picture matching task. The original studies, conducted at Purdue University and Midwestern University, examined the effects of music on attention in PWA (Purdue) and the relationship between attention and language in PWA (Midwestern). Forty-one PWA completed the Purdue study, while 17 PWA and the control group completed the procedures as part of the Midwestern University study.

Prior to pooling the two datasets, we evaluated potential between-site differences in demographic, clinical, and task-related variables. The samples did not differ significantly in age, aphasia severity (WAB-R Aphasia Quotient), or time post-stroke, although years of education differed across sites (Supplementary Table S1). To evaluate potential site-related differences in task performance, we compared reaction time and accuracy trajectories across datasets and found comparable patterns over the course of the experiment. Within-task reliability estimates were also similar across sites (Purdue ICC = .70; Midwestern ICC = .75). Variance decomposition using unconditional mixed-effects models further indicated that most variability was attributable to between-participant differences (ICC = .62), whereas a smaller proportion was associated with study site (ICC = .17) (Supplementary Table S2). Together, these analyses indicate that site contributed limited variance, supporting the pooling of the datasets. Nevertheless, because education differed across sites and was nested within site, study site was included as a fixed effect in the mixed-effects models to account for educational differences and other potential procedural or sampling variation differences between datasets.

### Attention Network Test (ANT)

**Figure 1.**
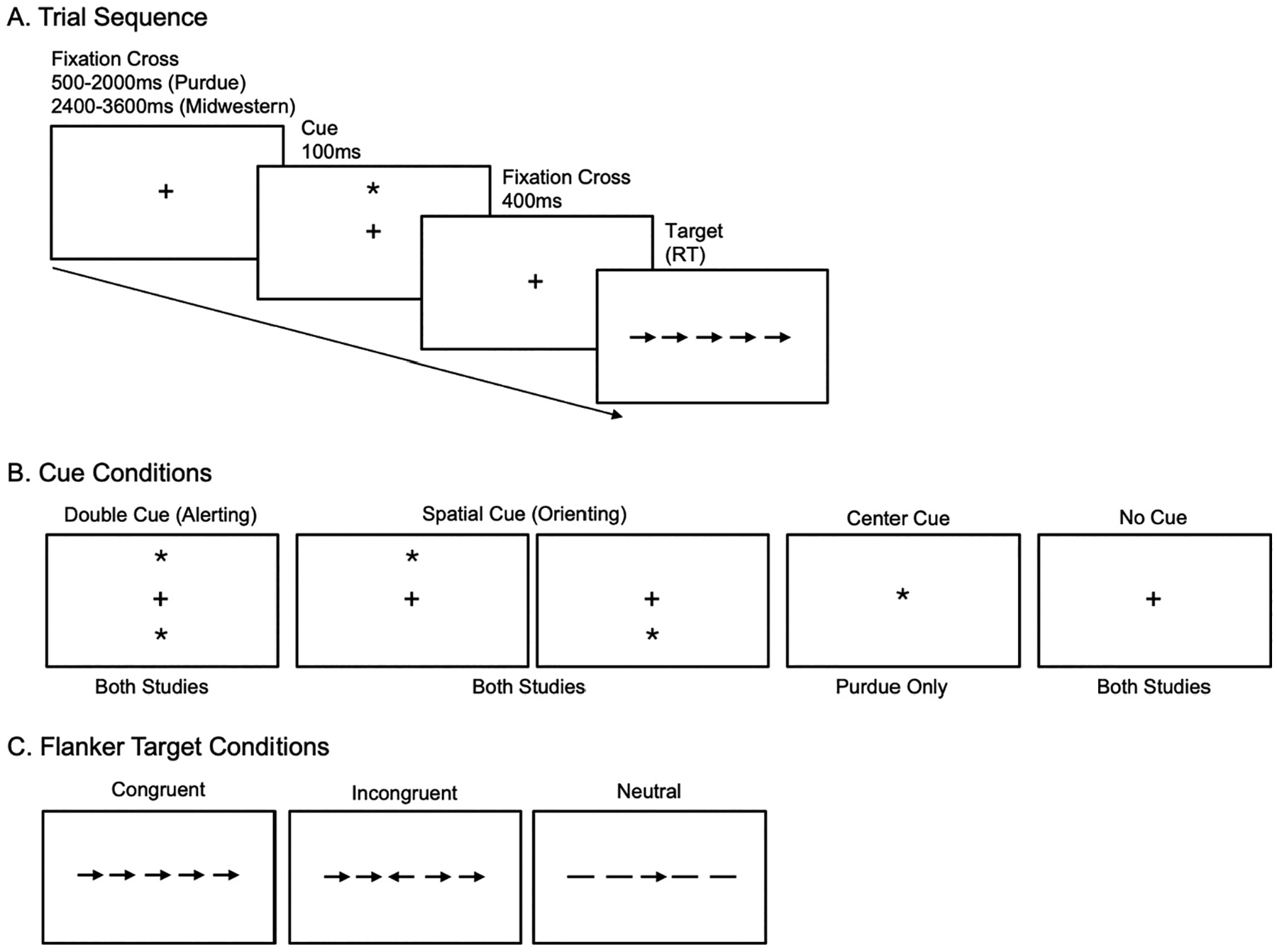
Attention Network Test trial sequence (A), cue conditions (B), and flanker targets (C).

Figure 1 includes a schematic of an ANT trial, as well as examples of the cue conditions and targets (Fan et al., 2002, 2009). Each trial began with a fixation cross jittered between 500–2000 (Midwestern) and 2400–3600 (Purdue) milliseconds. Following the offset of the fixation cross, participants were presented with a cue condition for 100 milliseconds (Figure 1B). Cue conditions for both studies included double cue (i.e., two asterisks presented simultaneously above and below the fixation cross), spatial cue (i.e., single asterisk presented above or below the fixation cross; spatial cues predicted the location of the upcoming flanker task with 75% accuracy), and no cue (i.e., no asterisk presented; fixation cross remained in the center of the screen). Participants in the Purdue study also saw a center cue (i.e., single asterisk presented in the center of the screen), however this condition was excluded from the analyses since it was not present in the Midwestern paradigm. Following the offset of the cue, participants were presented with a second fixation cross for 400 milliseconds, then the flanker task (Figure 1C).

In each flanker trial, participants were presented with a series of five stimuli consisting of a central target arrow flanked by either congruent arrows that face the same direction, incongruent arrows that face the opposite direction, or neutral flankers (two horizontal lines on each side) (Figure 1C). Participants were instructed to select which direction (left or right) the central arrow faced as quickly and accurately as possible. Reaction time and accuracy were recorded for each trial via keyboard button presses. To account for potential right-sided hemiparesis or limb apraxia in the aphasia group, all participants responded using their left hand. The “z” and “x” keys were labeled with left (<) and right (>) arrow stickers, respectively, to reinforce left-handed respondes.

Participants received verbal and written instructions and completed 5 practice trials prior to the onset of the experimental task. Purdue participants completed 156 experimental trials, while Midwestern participants completed 70 experimental trials. In line with the ANT literature (e.g., Fan et al., 2002), the efficiency of the alerting subsystem was computed by comparing reaction times on no cue and double cue trials, orienting by comparing reaction times on double cue and spatial cue trials, and executive control by comparing reaction times on incongruent and neutral trials.

### Sentence-picture matching task

The sentence stimuli have been described in detail in previous work (LaCroix et al., 2019; LaCroix, Blumenstein, et al., 2020; Wilson et al., 2010). Briefly, participants listened to two sentence structures spoken with a natural speech prosody. Each sentence was comprised of 10 syllables, two nouns (boy, girl), one of seven verbs (kick, wash, chase, push, kiss, pull, hug), and one of three colors (blue, green, red). The canonical sentences had a subject-relative structure (e.g., The boy who is blue is chasing the girl) and the non-canonical sentences had an object-relative structure (e.g., The girl who the boy is chasing is blue). The key difference between these two syntactic structures is that subject-relatives preserve English’s canonical subject-verb-object order within the embedded relative clause, whereas object-relatives disrupt this order within the relative clause by fronting the object (represented by the relative pronoun *who* for *the girl*), resulting in an apparent object-subject-verb sequence.

Each trial began with the simultaneous presentation of a binaurally presented sentence and two pictures (one target, one foil). Participants were instructed to decide which picture matched the sentence as quickly and accurately as possible. Reaction time and accuracy were collected for each trial via keyboard press (Purdue: “z” and “x” keys; Midwestern: “f” and “j” keys). To accommodate potential right-sided hemiparesis or limb apraxia in the aphasia group, all participants responded using their left hand. Verbal and written instructions preceded the start of the task. Participants in the Purdue study completed 5 practice trials followed by 70 experimental trials, of which 34 (17 canonical and 17 non-canonical) were included in the analysis. Participants in the Midwestern study completed 3 practice trials followed by 80 experimental trials, of which 20 (10 canonical and 10 non-canonical) were included in analyses.

### Statistical analyses

All data were analyzed using RStudio version 4.4.1 (R Core Team, 2025). All ANT trials (156 from Purdue and 70 from Midwestern) were included in the analyses, as restricting the Purdue dataset to the first 70 ANT trials per participant did not qualitatively change the results. Reaction times (RTs) for incorrect responses and those exceeding 2.5 standard deviations from each participant’s own mean were excluded. This within-participant trimming approach preserves between-participant differences in attention while removing only extreme deviations unlikely to reflect stable performance, such as very short or very long responses that are more likely to reflect transient disengagement, motor artifacts, or technical errors rather than true attentional performance (e.g., Thériault et al., 2024). Following this approach, 2.41% of trials were excluded for controls (.004% errors, 2.41% long RTs) and 1.62% of trials for PWA (.02% errors, 1.60% long RTs). The remaining trials, consisting of correct responses only, were used in the analyses.

### Impact of aphasia severity on attention: between group differences

From the remaining reaction time data, we computed RT difference scores for each participant as follows: alerting (no cue – double cue), orienting (double cue – spatial cue), and executive control (incongruent – neutral trials). These difference scores are consistent with the ANT literature (Fan et al., 2002), and are designed to isolate attention-specific effects from individual differences in overall reaction time or processing speed, thereby enabling more meaningful between-group comparisons. We conducted three separate ANOVAs, one for each attentional subsystem. In all models, the dependent variable was the RT difference score and the independent variable was aphasia severity (control, latent, mild, moderate, severe).

### Impact of aphasia severity on attention: within group effects

We additionally investigated whether the alerting (no cue vs. double cue), orienting (double cue vs. spatial cue), and executive control (incongruent vs. neutral trials) effects were present *within* each group. To do this, we computed a linear mixed-effects model using the lmer function from the lme4 package (Bates et al., 2015). The dependent variable was the log-transformed reaction time, which was applied to meet model assumptions of variance homogeneity. The fixed effects were cue (no cue, double cue, spatial cue), congruency (congruent, incongruent, neutral), and aphasia severity (control, latent, mild, moderate, severe). Study site (Purdue, Midwestern) was included as a fixed-effect covariate to account for differences in years of education and other potential site-related differences between datasets. Aphasia severity was categorical and based on the WAB-R Aphasia Quotient (WAB-AQ). We also specified the cue × aphasia severity and congruency × aphasia severity interactions, as these would allow us to investigate the presence of each effect *within* each group. Subject was specified as a random intercept to account for individual variability in response times. The results were first summarized as an Analysis of Deviance using car:Anova (Fox & Weisberg, 2019). Pairwise comparisons for the fixed and interaction effects were computed using “emmeans” (Length, 2023). Multiple corrections were corrected using the Benjamini-Hochberg (BH) procedure (Benjamini & Hochberg, 1995).

### Relationship between attention, aphasia severity, and sentence comprehension

Only participants with aphasia were included in the following analyses. Our primary analyses used structural equation modeling, implemented in lavaan (Rosseel, 2012), to test whether aphasia severity statistically mediated the relationship between attention and sentence comprehension. The three attention measures (RT difference scores for alerting, orienting, and executive control) were specified as exogenous predictors, and aphasia severity (indexed by the WAB-AQ) was included as the mediator. Sentence comprehension, operationalized as BIS efficiency scores for canonical and non-canonical sentences, served as the outcome variable. All direct paths were specified, and the residual variances of canonical and non-canonical BIS scores were allowed to covary to account for shared method variance. Standard errors were estimated using nonparametric bootstrapping with 5,000 draws. Both unstandardized and standardized path coefficients, along with 95% bootstrap confidence intervals, were reported.

Sentence comprehension was operationalized using a Balanced Integration Score (BIS; Liesefeld & Janczyk, 2019), which combines accuracy and response time into a single efficiency metric that accounts for potential speed-accuracy tradeoffs. We elected to use the BIS rather than modeling accuracy and response time separately in parallel models, as these measures capture overlapping aspects of performance and BIS provides a parsimonious representation of both in a single score. BIS was computed by z-transforming accuracy and reaction time across participants and subtracting standardized reaction time from standardized accuracy. Because BIS is derived from standardized values, it is centered at zero, with higher values reflecting greater accuracy and faster responses. The BIS approach was not applied to the ANT indices because, unlike the sentence-picture matching task, ANT accuracy was near ceiling (98.18 ± 13.36% across PWA and 98.69 ± 11.39% in controls); thus, incorporating accuracy into a composite score would have contributed minimal additional information beyond the RT-based attentional contrasts.

We additionally conducted exploratory Pearson correlations, specified with cor.test (R Core Team, 2025), to characterize associations between attention and language measures beyond sentence comprehension. The WAB-R subtests were not included as outcome variables in the structural equation model to avoid statistical circularity, as they contribute directly to the WAB-AQ mediator. Alerting, orienting, and executive control attention were each represented by the RT difference score and separately correlated with aphasia severity (WAB-AQ), each subtest of the WAB-R (spontaneous speech, auditory comprehension, repetition, naming/word finding), and canonical and non-canonical sentence comprehension.

## Results

### Impact of aphasia severity on attention: between group differences

**Figure 2.**
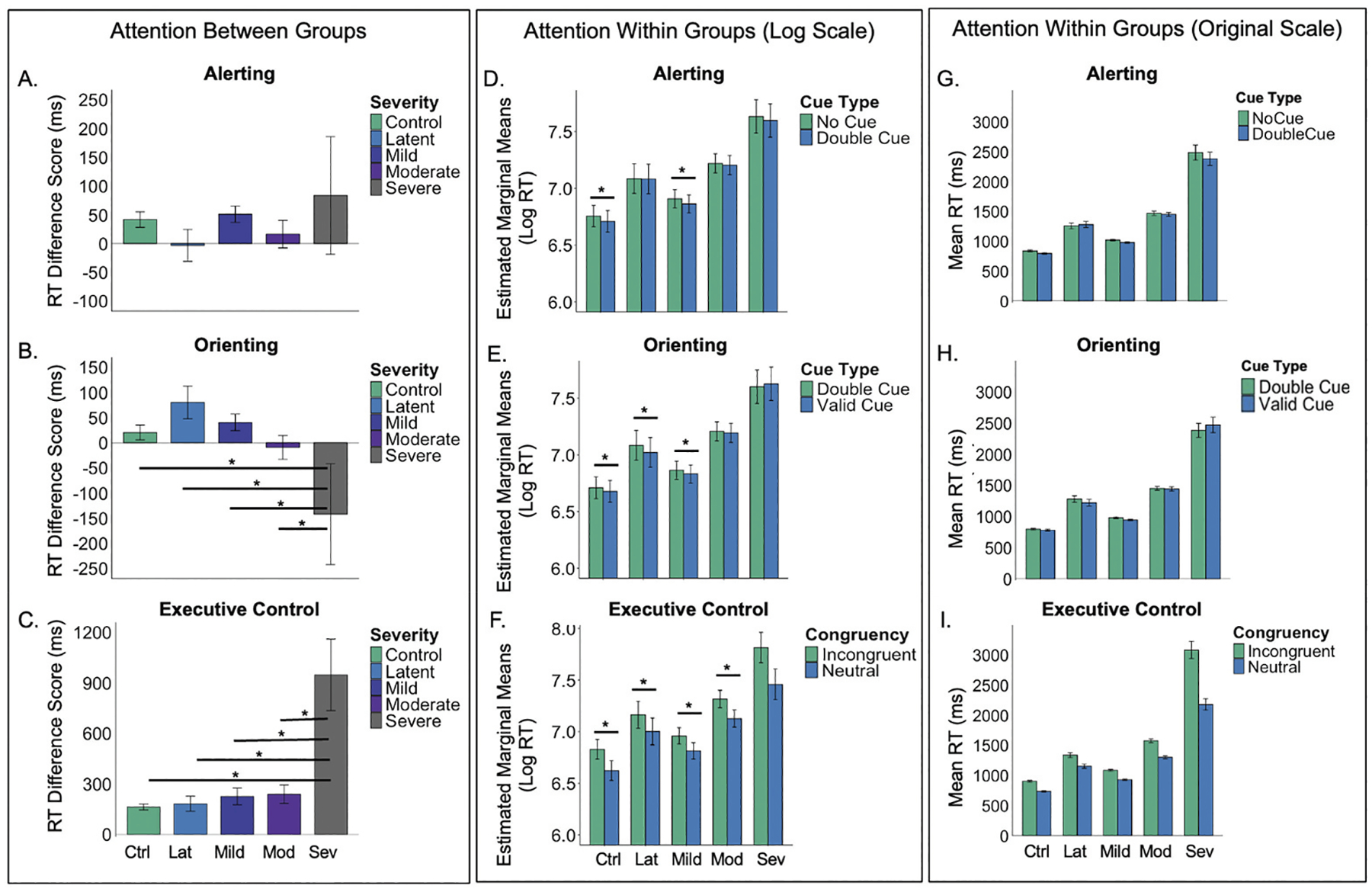
The left panel shows between-group differences in attention, represented as RT difference scores for each subsystem: (A) alerting = no cue – double cue, (B) orienting = double cue – spatial cue, and (C) executive control = incongruent – neutral targets. Larger positive values indicate faster responses to the alerting (double) cue, orienting (spatial) cue, and neutral flanker target. The middle panel presents estimated marginal means showing comparisons for attention within each group: (D) alerting (no cue vs. double cue), (E) orienting (double cue vs. spatial cue), and (F) executive control (incongruent vs. neutral). The right panel presents mean RTs showing comparisons for attention within each group: (G) alerting, (H) orienting, and (I) executive control. Error bars represent ±1 SD. Note. ms = milliseconds, Ctrl = control, lat = latent, mod = moderate, sev = severe.

The overall ANOVA models demonstrated that orienting, F(4, 74) = 4.70, *p* = .002, and executive control attention, F(4, 74) = 14.19, *p* < .001, differed by aphasia severity, but that alerting attention did not, F(4, 74) = .95, *p* = .44. All pairwise comparisons are reported in Table 2 and graphed in Figure 2. The severe aphasia group had poorer orienting (Figure 2B) and executive control attention (Figure 2C) than all other groups, but no other group differences were observed.

### Impact of aphasia severity on attention: within group effects

The full model results are reported in Table 3. The fixed effect of cue and congruency were both significant. As expected, all participants were slower on the no cue than double cue trials (the alerting effect), double cue than spatial cue trials (the orienting effect), and incongruent compared to neutral trials (the executive control effect). The fixed effect of aphasia severity^1^ was also significant, as were the aphasia severity × cue and aphasia severity × congruency interactions, which allowed us to explore the presence of each attentional effect within each group. As reported in Table 4 and depicted in Figure 2D–I, the control group demonstrated all three effects in the expected direction (i.e., better performance on double cue, spatial cue, and neutral trials), as did the mild aphasia group.

However, the latent aphasia group only demonstrated the orienting and executive control effects, but not the alerting effect, whereas the moderate and severe aphasia groups demonstrated the executive control effect only.

Relationship between attention, aphasia severity, and sentence comprehension

### Structural equation model

**Figure 3.**
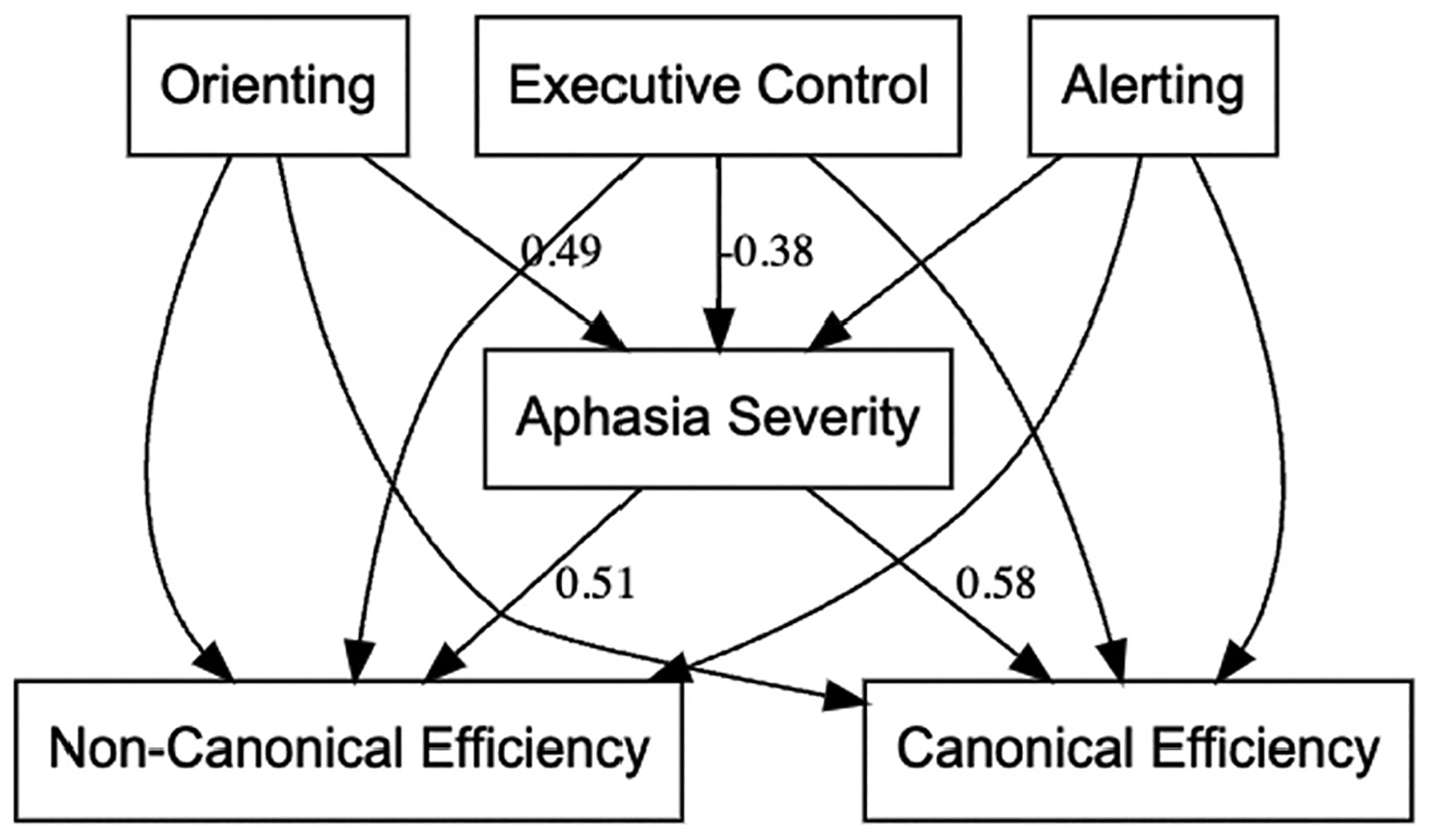
Mediation model illustrating the relationships among the attentional subsystems, aphasia severity, and sentence comprehension efficiency. All model paths tested are displayed; only standardized coefficients significant at *p* < .10 are labeled. Better orienting and executive control were associated with less severe aphasia, which in turn predicted greater efficiency for both canonical and non-canonical sentence comprehension. Alerting was not significantly related to aphasia severity or sentence comprehension efficiency.

The full model results are presented in Supplementary Table S4 and illustrated in Figure 3. Orienting (β = .49, *p* = .001) and executive control (β = −.38, *p* = .019) predicted aphasia severity, such that stronger attention was associated with milder aphasia. In turn, aphasia severity predicted both canonical (β = .58, *p* = .003) and non-canonical efficiency (β = .51, *p* = .012), with milder aphasia linked to more efficient sentence processing. The direct effects from alerting, orienting, and executive control to sentence comprehension efficiency were all non-significant. The indirect effects confirmed that orienting attention influenced both canonical (β = .28, *p* = .019) and non-canonical sentence comprehension efficiency (β = .25, *p* = .032) through aphasia severity, while executive control showed trend-level indirect effects (*p* < .10). Collectively, the mediation analysis indicates that the effect of orienting attention on sentence processing in PWA is fully mediated by aphasia severity. In other words, orienting supports comprehension indirectly by reducing aphasia severity, rather than exerting a direct influence on sentence processing efficiency.

### Correlations

**Figure 4.**
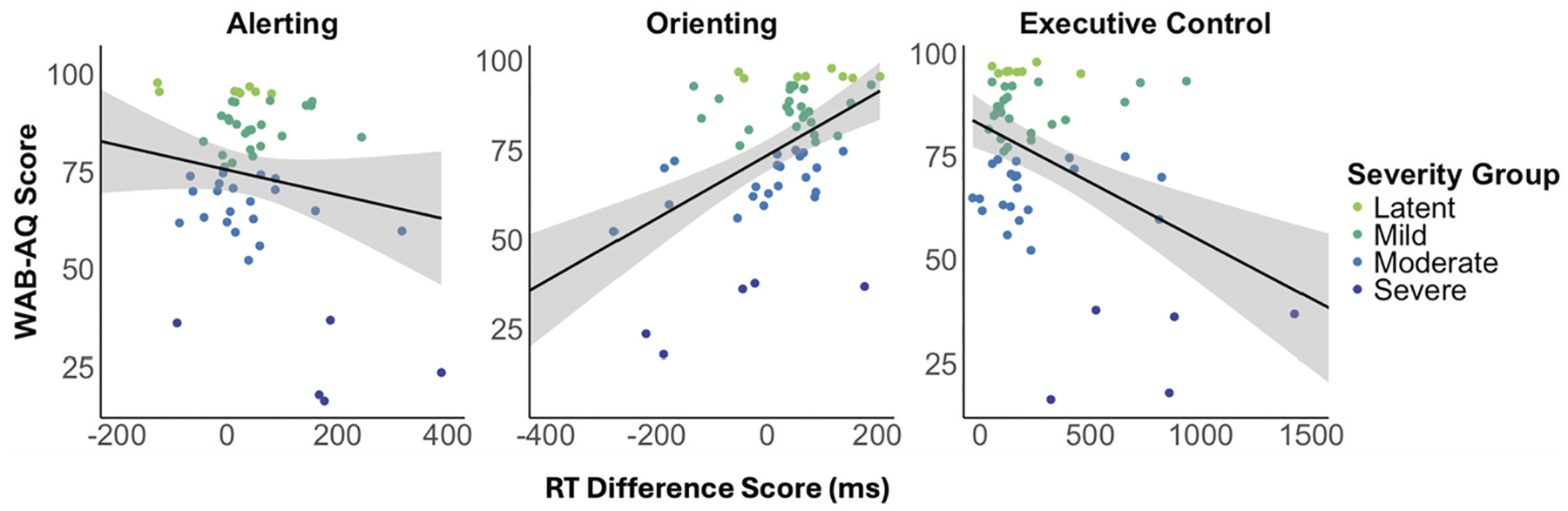
Scatterplots depicting the correlations between each attentional subsystem and aphasia severity (WAB-AQ). Larger more positive scores equate to faster performance following the alerting (double) cue, orienting (spatial) cue, and neutral flanker targets. Confidence intervals are +/− 95%.

Alerting attention was negatively correlated with the efficiency of canonical sentence comprehension, indicating that PWA who responded more quickly to the double cue showed less efficient canonical sentence processing (Table 5). Alerting attention did not correlate with aphasia severity (Figure 4, left panel), or any other language measure. Orienting attention was positively correlated with aphasia severity (Figure 4, center panel), all four WAB-R subtests, and canonical sentence comprehension efficiency, such that faster responses to the spatial cue were associated with less severe aphasia, milder language deficits overall, and more efficient canonical sentence processing. Executive control was negatively correlated with aphasia severity (Figure 4, right panel), all four WAB-R subtests, and canonical sentence comprehension efficiency, indicating that better executive control was linked to less severe aphasia, milder language deficits, and more efficient canonical sentence processing. Non-canonical sentence comprehension efficiency did not correlate with any attention measure.

## Discussion

The relationship between attention and language in aphasia is not always clear, with some studies reporting associations (Dutta et al., 2024; Fitzhugh et al., 2021; Frankel et al., 2007; Harmon et al., 2024; Huang et al., 2022; Kalbe et al., 2005; LaCroix, Blumenstein, et al., 2020; Nelson et al., 2023; Pérez Naranjo et al., 2018; Villard & Kidd, 2019) and others finding none (Gordon-Pershey & Wadams, 2017; Murray et al., 1997; Yao et al., 2020). Aphasia severity, however, is consistently linked to both attention and language (Fonseca et al., 2019; Lee et al., 2020; Meier et al., 2022; Murray, 2012; Schumacher et al., 2022), suggesting that severity may link these processes and help explain the mixed findings in the literature. To this end, we examined how domain-general attention deficits vary as a function of aphasia severity and tested whether severity statistically mediates the relationship between attention and sentence comprehension.

### Attention and aphasia severity

Alerting attention did not differ significantly across groups and showed no relationship with aphasia severity. The absence of an association with severity is consistent with previous findings (Huang et al., 2022), yet the lack of group differences contrasts with prior reports of reduced alerting in PWA compared to controls (Dash et al., 2020; Korda & Douglas, 1997; LaCroix, Blumenstein, et al., 2020; Laures, 2005; Mohapatra & Dash, 2023). While these group-level results suggest that alerting may be relatively preserved in aphasia and not systematically tied to severity, the within-group analyses provide additional nuance: the alerting effect (double < no cue) was only significant in the control and mild aphasia groups.

The absence of an alerting effect in the latent, moderate, and severe aphasia groups may reflect generalized slowing as all three groups showed markedly slower response times than the control group (Supplementary Table S3). Such slowing may reduce sensitivity to phasic alerting cues, which operate on a relatively brief timescale (<500 ms) (Fan et al., 2002; Hackley, 2009). In other words, when baseline processing speed is substantially delayed, participants may be unable to take advantage of the transient benefit provided by an alerting cue, even if the underlying neural mechanisms for alerting remain intact. This account highlights the importance of considering general processing speed when evaluating attention in aphasia, as slowed responses may mask or diminish the appearance of otherwise preserved attentional effects.

An alternative explanation is that procedural differences across studies, specifically variation in jitter duration (shorter: 500–2000 ms vs. longer: 2400–3600 ms), may have influenced alerting magnitude. Because alerting is sensitive to temporal expectancy and tonic readiness, longer jitter intervals may promote increased vigilance by allowing anticipation for cue onset to build more over time, potentially enhancing the magnitude of the alerting effect (Correa & Nobre, 2008; Correa et al., 2005; Nobre & van Ede, 2018). However, several observations suggest that jitter timing is unlikely to explain the present findings. Reliable alerting effects occurred under both timing protocols: all control participants were tested with the longer jitter interval, whereas the mild aphasia group – most of whom were tested with the shorter interval – also demonstrated a significant alerting effect. In addition, study site did not account for significant variance in unconditional mixed-effects models (Supplementary Table S2). Thus, these findings suggest that the absence of an alerting effect in the latent, moderate, and severe aphasia groups is unlikely to be attributable to jitter timing. Instead, the pattern more plausibly reflects generalized slowing, potentially arising from lesion-related disruption to neural systems supporting attention. Future work integrating lesion mapping and network-level connectivity analyses will be critical for clarifying how structural damage and large-scale network disruption contribute to altered alerting responses in subgroups of aphasia.

An emerging body of research has documented orienting attention impairments in post-stroke aphasia (Huang et al., 2022; LaCroix, Tully, et al., 2020; Petry et al., 1994; Robin & Rizzo, 1989; Villard & Kiran, 2015). The present findings are consistent with this literature and extend it by showing that orienting deficits scale with aphasia severity. Specifically, the orienting effect (spatial < double) was absent in the moderate and severe groups but present in the latent and mild aphasia groups. Moreover, most participants with severe aphasia (5/6) responded more slowly to the spatial cue than to the double cue, suggesting that the spatial cue may interfere with performance – a reversal previously observed in accuracy (LaCroix, Tully, et al., 2020). By contrast, most participants with latent aphasia (6/8) demonstrated the expected orienting effect. Although these patterns should be interpreted cautiously given the small sample sizes in the latent and severe groups, they suggest that orienting attention may remain relatively preserved when lesions are smaller or more localized (and/or potentially subcortical), but becomes increasingly compromised with greater lesion extent, particularly when frontal and parietal cortices are involved. However, this interpretation remains tentative because neural data were not directly collected in the present study, and lesion characteristics were not systematically measured but instead inferred indirectly from medical records and aphasia severity.

The executive control effect (incongruent < neutral) was significant in all groups and correlated positively with aphasia severity, such that individuals with more severe aphasia showed disproportionately larger interference costs. This pattern is consistent with prior evidence that executive control deficits worsen with increasing severity (Huang et al., 2022; Meier et al., 2022; Murray, 2012). Notably, only the severe group differed from controls, which may help reconcile mixed findings in the literature. Studies reporting no group differences have primarily sampled from mild-to-moderate participants (Dash et al., 2020; LaCroix, Blumenstein, et al., 2020; Mohapatra & Dash, 2023), whereas those identifying deficits included more individuals with severe aphasia (Huang et al., 2022). However, the relatively small number of participants with severe aphasia in the present sample warrants cautious interpretation, and replication in larger samples will be necessary to confirm the stability of this pattern. Regardless, these findings suggest that executive control deficits may not be a uniform feature of aphasia but instead become more pronounced as aphasia severity increases, underscoring the importance of considering severity when characterizing domain-general attention impairments in this population.

### Attention, sentence comprehension, and aphasia severity

Alerting attention was not significant in any path of the mediation model, despite its bivariate associations with aphasia severity and canonical sentence comprehension, suggesting that alerting does not influence sentence comprehension through a severity-mediated pathway. At the bivariate level, smaller alerting effects (i.e., similar performance across no cue and double cue trials) were associated with more efficient canonical sentence comprehension. Although larger alerting effects are typically interpreted as reflecting greater benefit from external cues (Fan et al., 2002), a smaller alerting effect may instead indicate more stable baseline attentional readiness. Individuals with higher baseline alertness may require less external cueing because they are already prepared to process incoming linguistic input. Thus, alerting attention may function as a background or modulatory influence on sentence comprehension by supporting stable readiness to process linguistic information.

In contrast to alerting, both orienting and executive control attention demonstrated severity-mediated relationships with sentence comprehension: individuals who oriented more efficiently to spatial cues and who were less distracted by incongruent flankers exhibited less severe aphasia, which in turn was associated with more efficient comprehension of both canonical and non-canonical sentences. Thus, the SEM findings extend prior evidence linking orienting and executive control attention to language outcomes in post-stroke aphasia (Fitzhugh et al., 2021; Huang et al., 2022; Kalbe et al., 2005; LaCroix, Blumenstein, et al., 2020; Villard & Kidd, 2019) by demonstrating that their association with sentence comprehension operates indirectly through aphasia severity rather than exerting a direct effect.

An apparent discrepancy emerged when comparing the bivariate correlations and SEM results for the non-canonical sentences. Although the mediation analyses revealed indirect relationships between orienting and executive control attention and non-canonical sentence comprehension, attention was not related to non-canonical comprehension at the bivariate level. This pattern suggests that attention relates to non-canonical comprehension primarily through its association with aphasia severity. However, in the present sample, participant subtype was largely clustered within severity: the moderate and severe groups were comprised primarily of individuals with Broca’s and conduction aphasia, whereas the mild group was largely composed of individuals with anomic aphasia. This clustering raises the possibility that the observed severity effects may partly reflect agrammatic comprehension, or greater difficulty with non-canonical relative to canonical structures, which is more commonly associated with Broca’s and conduction aphasia (Baldo et al., 2008; Caplan et al., 2013; Caramazza & Zurif, 1976; Caramazza et al., 1981; Grodzinsky, 2000; Gvion & Friedmann, 2012; Love et al., 2008). It is therefore possible that performance on non-canonical sentences was primarily constrained by structural grammatical impairment, leaving limited opportunity for variability in attentional efficiency to exert an independent influence on sentence processing.

Despite this potential subtype confound, the broader pattern of bivariate associations suggests that attention covaries with language performance, as orienting and executive control were related to multiple subdomains of the WAB-R. Although these findings should be interpreted cautiously, these cross-domain associations provide preliminary evidence that attentional capacity may relate to language performance beyond sentence comprehension. This interpretation is consistent with capacity-based theories of sentence comprehension, which propose that domain-general attention contributes to syntactic performance by facilitating the efficient access, maintenance, and integration of linguistic representations (e.g., Cutter et al., 2022; Just et al., 1996; Meng & Bader, 2025).

In line with capacity-based perspectives, non-canonical constructions impose greater demands on thematic role assignment and structural reanalysis. These structural demands can exceed an individual’s available cognitive resources, effectively creating a bottleneck at the level of linguistic representation. Under such conditions, variability in attentional efficiency may be overshadowed by structural overload, potentially masking any influence of attentional capacity on sentence processing and helping to explain the absence of bivariate correlations between attention and non-canonical comprehension. In contrast, canonical sentences place lower demands on thematic analysis, allowing variability in domain-general attention to exert a more detectable influence at the bivariate level because structural processing demands are reduced.

Importantly, the SEM results indicate that for both sentence types, these relationships are accounted for by overall aphasia severity, suggesting that domain-general attention relates to sentence processing through its broader association with language system functioning (as indexed by the WAB-R) rather than through a syntax-specific pathway.

Overall, these findings indicate that attention relates to sentence comprehension primarily through its association with aphasia severity. Of the three attention networks, orienting and executive control were associated with sentence comprehension performance, with additional bivariate correlations observed across WAB-R subdomains, whereas alerting was less systematically related to these measures, suggesting it may function more as a background support mechanism. Although bivariate associations with WAB-R subscores suggest that attention may relate to performance across multiple language domains, these findings are correlational and should be interpreted as preliminary, requiring confirmation in future work. Importantly, these findings do not imply that aphasia is primarily an attentional disorder. Aphasia remains fundamentally a language impairment, with semantic and syntactic deficits central to sentence comprehension difficulties.

### Limitations and future directions

While informative, a few limitations warrant consideration. First, the sample size within individual severity groups – particularly latent and severe aphasia – was relatively small. This limits statistical power and may have contributed to variability in certain effects, including the absence of an alerting effect in the latent and severe groups. Replication in larger samples will be necessary to confirm the observed severity-dependent patterns.

Second, to enhance statistical power and ensure representation across aphasia severities, we combined data from two related studies. Although this approach increased the sample size, it also introduced minor methodological differences between datasets. These differences were accounted for by including study site as a fixed-effect covariate in the analyses. Nevertheless, pooling datasets may introduce some methodological heterogeneity, and future research should replicate these findings using a single, standardized protocol.

Third, structural and functional neuroimaging data were not collected as part of this study. Although medical records provided general information about neural damage, the absence of neuroimaging prevented us from directly linking attentional differences to lesion location or network-level disruption. Future studies incorporating structural and functional imaging will be critical for clarifying the neural mechanisms underlying attentional differences in aphasia.

Finally, although orienting and executive control were indirectly related to sentence comprehension through aphasia severity, these findings reflect statistical mediation within a cross-sectional design and therefore do not establish causal direction. The present data cannot determine whether reduced attentional efficiency contributes to greater aphasia severity, whether language impairment constrains attentional functioning, or whether both arise from shared neural disruption. Clarifying the nature and direction of this relationship will require longitudinal and lesion-informed studies that track changes in attention and language over time.

### Conclusions

This study demonstrates that attentional profiles in aphasia vary systematically with severity and that the relationship between orienting and executive control and sentence comprehension is accounted for by aphasia severity. By situating attention within the broader construct of aphasia severity, these findings help reconcile mixed results in the literature and clarify why attentional abilities may appear inconsistent across samples with differing impairment levels. The results further support the use of the ANT as a largely nonverbal index of attentional functioning in PWA. The present findings are also consistent with theoretical accounts proposing that domain-general attention may constrain sentence processing, although the cross-sectional nature of the data prevents conclusions about causal direction. Future research should therefore examine the directionality of this relationship, including whether attentional capacity constrains sentence comprehension or whether both attention and sentence comprehension impairments arise from shared network disruption.

## Supplementary Material

Supp 1

## Figures and Tables

**Table 1.** Participant demographic and study specific data.

Subject	Gender (F/M)	Age	Race	Eth.	Ed. (Yr.)	TPS (Mo.)	WAB-AQ	Aphasia Type and Severity
1002	F	49	White	Not H/L	17	134	97.8	Latent
1013	F	60	B/A	Not H/L	21	82	96.8	Latent
1015	F	43	White	Not H/L	17	27	95.6	Latent
0024	F	55	White	H/L	16	98	95.5	Latent
1007	M	58	White	Not H/L	17	31	95.5	Latent
1039	M	76	White	Not H/L	17	96	95.4	Latent
1018	M	66	White	Not H/L	21	45	95.1	Latent
0006	M	44	White	NR	16	63	95.0	Latent
0017	M	65	White	Not H/L	20	107	93.2	Mild Anomic
0014	F	57	White	Not H/L	18	67	93.0	Mild Anomic
0001	M	31	NR	H/L	14	52	93.0	Mild Anomic
1019	F	56	B/A	Not H/L	19	438	92.8	Mild Anomic
1041	M	67	White	Not H/L	19	37	92.0	Mild Anomic
1009	M	63	White	Not H/L	19	146	91.9	Mild Anomic
1014	F	51	White	Not H/L	19	281	89.3	Mild Anomic
0027	M	59	White	Not H/L	12.5	6	88.6	Mild Anomic
0030	F	72	White	H/L	9	81	88.1	Mild Anomic
1045	F	49	White	Not H/L	17.5	116	87.1	Mild Anomic
1033	F	50	White	Not H/L	17	47	87.0	Mild Anomic
1010	M	58	White	H/L	21	46	85.7	Mild Anomic
1040	F	63	White	Not H/L	19	62	85.6	Mild Anomic
1038	M	55	B/A	Not H/L	17	68	84.8	Mild Anomic
1035	M	59	White	Not H/L	17	86	84.1	Mild Anomic
0002	F	49	White	Not H/L	12	91	83.8	Mild Anomic
1025	M	61	Asian	Not H/L	21	137	82.7	Mild Anomic
1006	M	63	White	Not H/L	17	72	81.5	Mild Conduction
1030	M	71	White	Not H/L	19	80	80.6	Mild Anomic
1029	F	61	White	Not H/L	19	33	79.2	Mild Broca’s
1028	M	62	White	Not H/L	19	237	78.9	Mild Anomic
1047	M	72	White	Not H/L	13	179	77.2	Mild Anomic
1037	M	67	B/A	Not H/L	19	36	76.2	Mild Anomic
1042	M	59	B/A	Not H/L	13	61	74.9	Moderate Broca’s
1043	M	54	White	Not H/L	17	94	74.6	Moderate Anomic
1023	F	61	Asian	Not H/L	19	104	74.2	Moderate Wernicke’s
1022	M	70	White	Not H/L	17	35	74.2	Moderate Transcortical Motor
1001	F	52	White	Not H/L	17	84	73.8	Moderate Conduction
0031	F	77	White	Not H/L	12	120	73.2	Moderate Conduction
1052	F	45	White	Not H/L	17	97	70.7	Moderate Conduction
1044	M	57	White	Not H/L	19	87	70.3	Moderate Conduction
1012	M	82	White	Not H/L	19	39	70.0	Moderate Conduction
0025	F	49	White	Not H/L	12	20	69.9	Moderate Transcortical Motor
1032	M	46	White	Not H/L	17	96	67.3	Moderate Broca’s
0009	M	63	White	H/L	14	176	64.9	Moderate Broca’s
0012	F	73	White	H/L	18	74	64.7	Moderate Broca’s
1011	M	65	White	Not H/L	15	60	63.2	Moderate Broca’s
1036	M	67	White	Not H/L	17	49	62.8	Moderate Broca’s
1021	M	46	White	Not H/L	17	42	62.0	Moderate Broca’s
1046	M	70	White	Not H/L	13	43	61.8	Moderate Wernicke’s
0032	F	65	White	Not H/L	12	27	59.7	Moderate Transcortical Motor
1053	F	41	White	Not H/L	19	39	59.4	Moderate Broca’s
0018	F	49	White	Not H/L	16	31	55.9	Moderate Broca’s
1016	M	74	White	Not H/L	13	50	52.2	Moderate Wernicke’s
1027	M	66	White	Not H/L	17	35	37.7	Severe Broca’s
1034	M	59	White	Not H/L	15	103	36.8	Severe Broca’s
0029	M	59	White	Not H/L	16	13	36.1	Severe Broca’s
0022	F	58	White	Not H/L	18	129	23.6	Very Severe Broca’s
1024	M	66	White	Not H/L	16	73	17.9	Very Severe Broca’s
0028	F	64	White	Not H/L	15.5	7	16.3	Very Severe Broca’s
PWA Avg.	24/34	59.47	—	—	16.71	83.85	74.50	—
Control Avg.	10/21	57.14	—	—	15.62	—	—	—

Note. F = female; M = male; Eth. = ethnicity; Ed. = education; Yr. = years; TPS = time post-stroke; Mo = months; WAB-AQ = Western Aphasia Battery–Revised Aphasia Quotient; H/L = Hispanic/Latino; B/A = Black or African American; NR = no response; Avg. = average scores across all participants in each group. Subject identification numbers beginning with “1” were tested at Purdue University, whereas those beginning with “0” were tested at Midwestern University.

**Table 2.** Pairwise comparisons of alerting, orienting, and executive control attention across different levels of aphasia severity.

Comparison	Alerting	Orienting	Executive Control
Control vs. Latent	*t* = 1.05, *p* = .59	*t* = −1.37, *p* = .25	*t* = −.20, *p* = .85
Control vs. Mild	*t* = −.30, *p* = .77	*t* = −.63, *p* = .53	*t* = −.89, *p* = .63
Control vs. Moderate	*t* = .80, *p* = .61	*t* = .91, *p* = .41	*t* = −1.05), *p* = .60
Control vs. Severe	*t* = .80, *p* = .61	*t* = 3.33, *p* = .005*	*t* = −7.23, *p* < .001*
Latent vs. Mild	*t* = −1.29, *p* = .59	*t* = .92, *p* = .41	*t* = −.45, *p* = .82
Latent vs. Moderate	*t* = −.46, *p* = .72	*t* = 2.04, *p* = .09	*t* = −.58, *p* = .80
Latent vs. Severe	*t* = −1.57, *p* = .59	*t* = 3.91, *p* = .002*	*t* = −6.05, *p* < .001*
Mild vs. Moderate	*t* = 1.12, *p* = .59	*t* = 1.56, *p* = .21	*t* = −.18, *p* = .85
Mild vs. Severe	*t* = −.70, *p* = .61	*t* = 3.77, *p* = .002*	*t* = −6.72, *p* < .001*
Moderate vs. Severe	*t* = −1.42, *p* = .59	*t* = 2.72, *p* = .02*	*t* = −6.53, *p* < .001*

* Significant *p* < .05. Degrees of freedom equal 74 for all pairwise comparisons.

**Table 3.** Full model results for the LMER model.

Effect	Statistic	
Intercept	F(1, 73.1) = 17050.57, *p* < .001*	
Congruency	F(2, 6829.3) = 340.61, *p* < .001*	
Cue Condition	F(2, 6829.1) = 2.43, *p* < .001*	
Aphasia Severity	F(4, 73.0) = 9.5, *p* < .001*	
Study Site	F(1, 73.1) = 19.32, *p* = .123	
Congruency ×Aphasia Severity	F(8, 6829.2) = 6.91, *p* < .001*	
Cue Condition ×Aphasia Severity	F(8, 6829.1) = 2.22, *p*^†^= .023*	
Random Effects	Variance (SD)	ICC
Subject	.13 (.36)	.69
Residual	.06 (.24)	

* Significant *p* < .05.

† Model RT_Log_~cue*aphasia_severity+congruency*aphasia_severity+site+(1|subject).

R^2^_conditional_: .78; R^2^_marginal_: .30.

**Table 4.** Pairwise comparisons showing the significance of the alerting, orienting, and executive control effects within each group.

	Alerting	Orienting	Executive Control
Control	*t* = 3.03, *p* = .005*	*t* = 2.07, *p* = .04*	*t* = 13.35, *p* < .001*
Latent	*t* = .17, *p* = .87	*t* = 2.93, *p* = .007*	*t* = 7.68, *p* < .001*
Mild	*t* = 3.62, *p* = .001*	*t* = 2.57, *p* = .01*	*t* = 11.58, *p* < .001*
Moderate	*t* = 1.11, *p* = .33	*t* = .97, *p* = .33	*t* = 14.23, *p* < .001*
Severe	*t* = 1.29, *p* = .33	*t* = −.98, *p* = .33	*t* = 13.17, *p* < .001*

* Significant *p* < .05.

**Table 5.** Pearson correlations between each attentional subsystem and language variable.

	Alerting	Orienting	Executive Control
Aphasia Severity (WAB-AQ)	r = −.18, *p* = .17	r = .56, *p* < .001*	r = −.48, *p* < .001*
Spontaneous Speech	r = −.19, *p* = .15	r = .53, *p* < .001*	r = −.50, *p* < .001*
Auditory Verbal Comprehension	r = −.14, *p* = .31	r = .54, *p* < .001*	r = −.42, *p* < .001*
Repetition	r = −.14, *p* = .31	r = .48, *p* < .001*	r = −.38, *p* = .004*
Naming and Word Finding	r = −.17, *p* = .20	r = .53, *p* < .001*	r = −.44, *p* < .001*
Canonical BIS	r = −.32, *p* = .01*	r = .34, *p* = .009*	r = −.35, *p* = .007*
Non-Canonical BIS	r = −.08, *p* = .56	r = .09, *p* = .49	r = −.03, *p* = .81

* Significant *p* < .05. Degrees of freedom equal 56 for all correlations.

## Data Availability

The data that support the findings of this study are available from the corresponding author, upon reasonable request.

## Footnotes

1. The fixed effect of aphasia severity was significant. However, this effect likely reflects processing speed rather than attention, as it captures overall reaction time differences across groups rather than group differences based on the difference scores isolating each attentional subsystem, as reported in Table 2. Pairwise comparisons exploring this fixed effect are reported in Supplementary Table S3. Overall, the control group was faster than the latent aphasia group, the mild aphasia group was faster than the moderate aphasia group, and the moderate aphasia group was faster than the severe aphasia group. The latent aphasia group did not differ from the mild aphasia group.
