# Supplementary material for: Aphasia severity mediates the relationship between attention and sentence comprehension
Source: Aphasiology. Author manuscript; Available in PMC 2026 Jun 2. (PMC13225851; doi:10.1080/02687038.2026.2671291)
Supplement: Supp 1 [file NIHMS2178963-supplement-Supp_1.docx]

| **Supplementary Table 1.** Independent sample t-tests comparing demographic variables between PWA across the two study sites. | | | | | | | |
| --- | --- | --- | --- | --- | --- | --- | --- |
| Variable | Purdue | | Midwestern | |  |  |  |
|  | *M* | *SD* | *M* | *SD* | Statistic | | |
| Age | 60.00 | 9.44 | 58.18 | 11.50 | t(25.38) = .57, p = .57, d = .17 | | |
| Education | 17.38 | 2.21 | 14.41 | 2.86 | t(24.33) = 3.83, p = .001*, d = 1.11 | | |
| TPS (Months) | 90.41 | 77.60 | 68.35 | 48.40 | t(46.94) = 1.31, p = .20, d = .38 | | |
| WAB-AQ | 75.96 | 17.50 | 70.26 | 25.40 | t(22.58) = .85, p = .41, d =.24 | | |
| *Significant at p <.05  TPS = time post stroke | | | | | | | |

| **Supplementary Table 2.** Variance components from unconditional mixed-effects model partitioning variability into between-participant, between-site, and within-participant sources.  RT_Log_ ~ 1 + (1\|subject) + (1\|site) | | | |
| --- | --- | --- | --- |
|  | Variance | SD | ICC |
| Participant | .19 | .43 | .62 |
| Site | .05 | .28 | .17 |
| Residual (within participant) | .06 | .25 | -- |

| **Supplementary Table 3.** Pairwise comparisons for the fixed effect of aphasia severity from the LMER model. | | | | |
| --- | --- | --- | --- | --- |
|  | *B* | *SE* | *t* | *p* |
| Control vs. Latent  Control vs. Mild  Control vs. Moderate  Control vs. Severe  Latent vs. Mild  Latent vs. Moderate  Latent vs. Severe  Mild vs. Moderate  Mild vs. Severe  Moderate vs. Severe | -.35  -.15  -.49  -.91  .20  -.14  -.56  -.34  -.75  -.41 | .15  .11  .11  .18  .15  .15  .20  .11  .17  .17 | -2.30  -1.37  -4.32  -5.17  1.33  -.96  -2.83  -3.12  -4.50  -2.44 | .035*  .21  < .001*  < .001*  .21  .34  .01  .007*  < .001*  .03* |
| *Significant *p <* .05 | | | | |

| **Supplementary Table 4.** Full model results for the mediation model. | | | | | | |
| --- | --- | --- | --- | --- | --- | --- |
|  | Parameter | B | SE | β (Std.) | 95% CI | p |
| Direct Effect | Alerting → Aphasia Severity | 0.013 | 0.025 | 0.075 | –0.039, 0.059 | .593 |
|  | **Orienting → Aphasia Severity** | **0.077** | **0.022** | **0.491** | **0.023, 0.111** | **.001*** |
|  | **Executive Control → Aphasia Severity** | **–0.022** | **0.010** | **–0.382** | **–0.036, –0.002** | **.019*** |
|  | Alerting → Canonical Efficiency | –0.004 | 0.002 | –0.237 | –0.007, 0.001 | .067 |
|  | Orienting → Canonical Efficiency | –0.001 | 0.002 | –0.066 | –0.005, 0.004 | .680 |
|  | Executive Control → Canonical Efficiency | –0.000 | 0.001 | –0.019 | –0.001, 0.002 | .905 |
|  | Alerting → Noncanonical Efficiency | –0.001 | 0.002 | –0.094 | –0.005, 0.003 | .559 |
|  | Orienting → Noncanonical Efficiency | –0.002 | 0.002 | –0.174 | –0.006, 0.002 | .308 |
|  | Executive Control → Noncanonical Efficiency | 0.001 | 0.001 | 0.198 | –0.000, 0.003 | .266 |
|  | **Aphasia Severity → Canonical Efficiency** | **0.049** | **0.016** | **0.578** | **0.020, 0.084** | **.003*** |
|  | **Aphasia Severity → Noncanonical Efficiency** | **0.039** | **0.015** | **0.509** | **0.010, 0.071** | **.012*** |
| Residual Covariance | **Canonical Efficiency ~~ Non-Canonical Efficiency** | **1.191** | **0.336** | **0.660** | **—** | **< .001*** |
| Indirect Effects | Alerting → Aphasia Severity → Canonical Efficiency | 0.001 | 0.001 | 0.044 | –0.002, 0.004 | .639 |
|  | **Orienting → Aphasia Severity → Canonical Efficiency** | **0.004** | **0.002** | **0.284** | **0.001, 0.007** | **.019*** |
|  | Executive Control → Aphasia Severity → Canonical Efficiency | –0.001 | 0.001 | –0.221 | –0.002, 0.000 | .074 |
|  | Alerting → Aphasia Severity → Non-Canonical Efficiency | 0.001 | 0.001 | 0.038 | –0.002, 0.003 | .635 |
|  | **Orienting → Aphasia Severity → Non-Canonical Efficiency** | **0.003** | **0.001** | **0.250** | **0.001, 0.006** | **.032*** |
|  | Executive Control → Aphasia Severity → Non-Canonical Efficiency | –0.001 | 0.001 | –0.195 | –0.002, 0.000 | .097 |
| Total Effects (Direct + Indirect) | Alerting → Canonical Efficiency | -0.003 | 0.002 | -0.193 | -0.007, 0.001 | .167 |
|  | Orienting → Canonical Efficiency | 0.003 | 0.002 | 0.218 | -0.000, 0.007 | .100 |
|  | Executive Control → Canonical Efficiency | -0.001 | 0.001 | -0.24 | -0.003, 0.001 | .179 |
|  | Alerting → Non-Canonical Efficiency | -0.001 | 0.002 | -0.056 | -0.005, 0.004 | .729 |
|  | Orienting → Non-Canonical Efficiency | 0.001 | 0.002 | 0.076 | -0.002, 0.005 | .604 |
|  | Executive Control → Non-Canonical Efficiency | 0.0 | 0.001 | 0.003 | -0.001, 0.002 | .986 |
| Asterisk + bolding = significant at *p <* .05 | | | | | | |
